# Does Art Affect Nursing Students' Peace of Mind and Perceptions of Spiritual Care Giving in Türkiye?

**DOI:** 10.1007/s10943-025-02306-6

**Published:** 2025-04-28

**Authors:** Melike Taşdelen Baş, Birsel Molu, Funda Özpulat

**Affiliations:** Department of Nursing, Selcuk University Aksehir Kadir Yallagoz Health School, Konya, Turkey

**Keywords:** Spirituality, Nursing care, Emotions, Art, Nursing education

## Abstract

Nursing involves spiritual, physiological, psychological, and social care. Understanding the impact of art on nursing students’ perceptions of peace and spiritual care is essential. This descriptive study investigated how nursing students perceived peace and spiritual care after attending a decorative arts course. The research was conducted between June and July 2022 with a sample of 140 nursing students from a university in Türkiye. Data were collected using a personal information form, the Peace Scale, and the Spiritual Care-Giving Scale. Participants who had taken the “Decorative Arts” course for two terms had a significantly higher mean Peace Scale score than those who had taken it for one semester or had never taken it (*p* = .001).. Similarly, they had significantly higher mean scores on the Spiritual Care-Giving Scale. In particular, they scored higher on the ‘attributes for spiritual care,’ ‘spiritual care attitudes,’ and ‘spiritual care values’ subscales compared to those who had taken the course for one semester or had never taken it (*p* < .001). The “Decorative Arts” course positively influenced nursing students' perceptions of peace and spiritual care, highlighting the potential role of artistic engagement in nursing education.

## Introduction

Nurses must provide holistic, compassionate, dignified, and individualized care (Ross et al., 2018), as they care for individuals during the most vulnerable moments of their lives. Spiritual care is an integral part of holistic care (Lewinson et al., 2015). Spirituality is a set of values that connect individuals to themselves, their families, and their faith. It encompasses hope, belonging, commitment, health, and well-being. Spirituality helps individuals find meaning in life and provides strength to endure difficulties (Selman et al., 2018; Ross et al., 2014). Spiritual care is addressed in two main ways: first, by encouraging patients to maintain their existing religious and spiritual practices, and second, by providing therapeutic support, including companionship, active listening, exploration of life’s meaning, fostering hope, and other care-related interventions (Pesut, 2008).

Spiritual care includes various activities such as praying with patients, allowing them to express their faith and emotions, listening to them, and encouraging positive thinking (Kaddourah et al., 2018; Musa et al., 2017). It involves practices that meet patients' spiritual needs (Pesut, 2008) and is shaped by nurses’ attitudes of kindness, benevolence, compassion, and inner peace, which are often influenced by their spiritual experiences (Zare & Jahandideh, 2014; Timmins & Caldeira, 2017). Additionally, spirituality can help nurses achieve inner peace (Hamid & Dehghanizadeh, 2012).

Nursing offers an opportunity for healthcare professionals to incorporate spirituality into their practice. Professional education can enhance nurses’ awareness of spiritual care (Lewinson et al., 2015). However, research suggests that nursing curricula rarely address spiritual care adequately (Lewinson et al., 2015; Kalkım et al., 2018; Kroning, 2018). Kalkim et al. (2018) recommend revising nursing curricula, as students often lack competencies in spiritual care when they enter professional practice. Similarly, Kroning (2018) argues that nursing students feel uncomfortable providing spiritual care. Other studies also highlight that nursing students are not adequately prepared to deliver spiritual care in clinical practice (Minton et al., 2018; van Leeuwen et al., 2006; Williams et al., 2016).

Reading current research and theoretical articles can help nursing students develop a better understanding of spirituality and spiritual care (Smith & Read, 2017; Ross et al., 2016; Giske & Cone, 2015). Personal values, art, and meaningful stories can enhance individuals' awareness of spirituality (Smith & Read, 2017). Artistic materials and imagery help individuals make sense of spirituality, affirm their personality and identity, and perceive themselves as valuable. Art enables individuals to better understand themselves, strengthen their identity, and enrich their spiritual world. Through art, people can express their inner emotions and explore their spiritual journey more deeply. This process allows individuals to position themselves in a more meaningful way, both personally and socially (Gómez-Rincón, 2023; Sarman & Günay, 2023). Additionally, art can help healthcare professionals cope with emotionally challenging situations, develop self-awareness, and promote self-care (Potash et al., 2014; Huet, 2016).

Based on this perspective, we propose that an extracurricular art course may help nursing students develop a more positive perception of peace, spiritual care, and spirituality. Therefore, this study investigates how nursing students perceive spiritual care and their level of inner peace after attending a decorative arts course.

### Research Questions

- Does the "decorative arts" course affect nursing students' peace of mind?
- Does the "decorative arts" course influence nursing students' perceptions of spiritual caregiving?

## Methods

This descriptive study was conducted between June and July 2022. The study population consisted of 148 nursing students from a university in Türkiye. The final sample included 140 participants. A total of 140 students out of 148 completed the questionnaires, yielding a response rate of 94.6%.

**Fig. 1:**
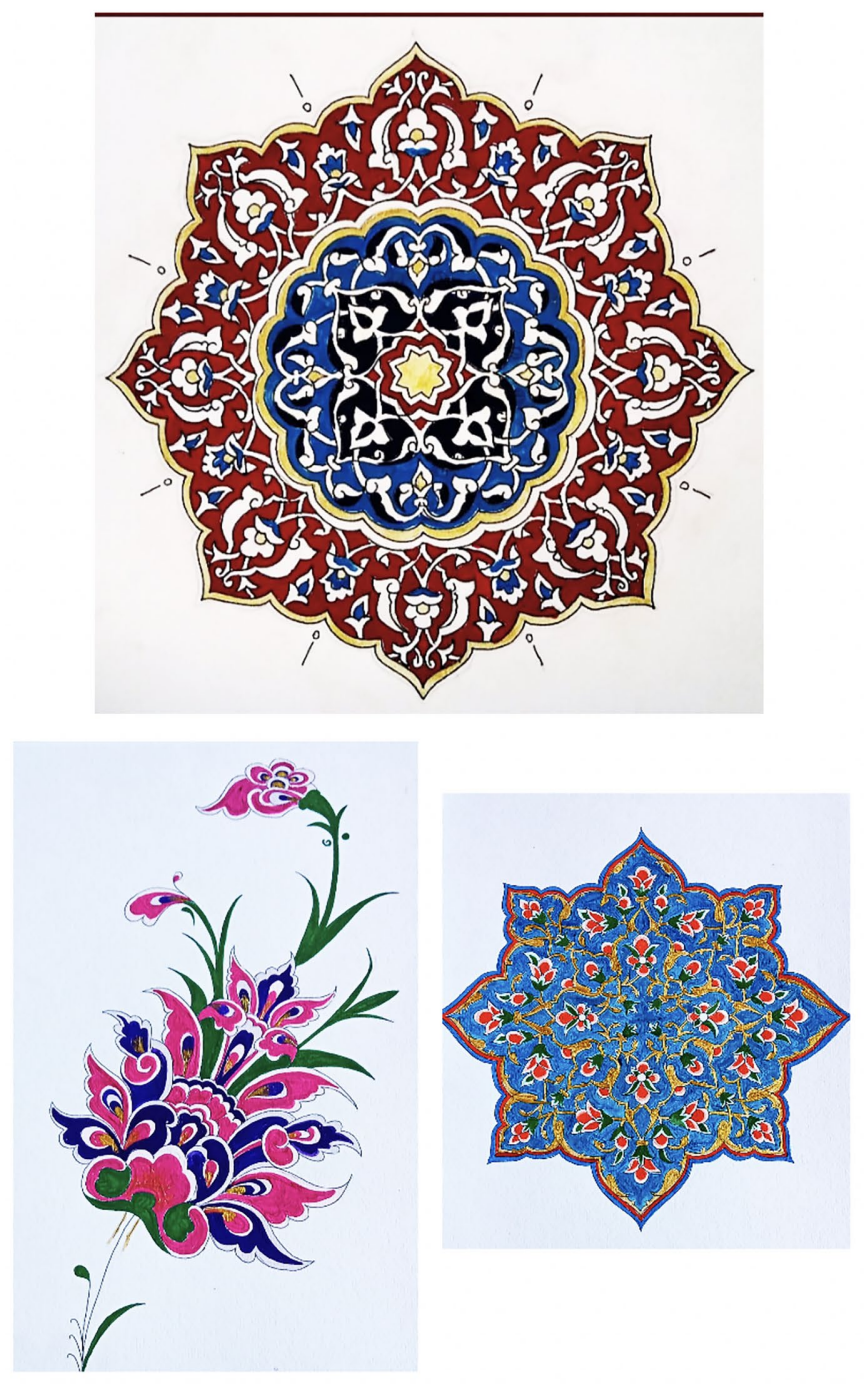
Pattern samples created by students attending the *“Decorative Arts”* course

The nursing department offers "**Decorative Arts**" as an elective course for third- and fourth-year students. Students can enroll in **Decorative Arts-1** (28 h) and **Decorative Arts-2** (28 h) over two semesters. The course covers **the Art of Illumination**, focusing on both traditional and modern motifs specific to illumination art, as well as drawing and coloring techniques applied to at least six different patterns. Three of the patterns selected by students for this semester are presented in Fig. 1. These patterns were created by students who participated in the course. The course is taught by an academic instructor certified in the Art of Illumination.

Data were collected online through a survey distributed to all participants via a digital link. This study was conducted in accordance with the principles of the **Declaration of Helsinki**.

Inclusion Criteria:

- Being a third- or fourth-year nursing student.

Exclusion Criteria:

- Not being a third- or fourth-year nursing student.

### Data Collection Tools

The data were collected using three instruments:

1. Personal Information Form
2. Peace Scale (PS)
3. Spiritual Care-Giving Scale (SCGS)

### Personal Information Form

The personal information form included ten items assessing participants' sociodemographic characteristics.

### Peace Scale (PS)

The **Peace Scale (PS)** was developed in Türkiye by Demirci and Ekşi (2017) to measure individuals' sense of peace. The scale consists of **eight items** rated on a **five-point Likert-type scale**. Construct validity was confirmed through **exploratory factor analysis**, with an eigenvalue of **3.226**, explaining **40.328%** of the total variance. The scale demonstrated high internal consistency, with a **Cronbach’s alpha reliability coefficient of 0.83**. In this study, the PS was used to assess nursing students' overall sense of peace. Higher scores indicate a greater sense of inner peace and emotional balance (Demirci & Ekşi, 2017).

*Example Items*:

- *"I generally feel at peace."*
- *"Despite the challenges I face, I can maintain balance in my life."*
- *"I am at peace with myself."*

### Spiritual Care-Giving Scale (SCGS)

The psychometric validation and translation of religious and spiritual measures play a critical role in determining the cultural adaptation and reliability of such measures (Koenig & Al Zaben, 2021). In this context, the Spiritual Care-Giving Scale (SCGS), originally developed by Tiew and Creedy (2012) to evaluate nursing students' perceptions of spirituality and spiritual care, was later adapted into Turkish by İpek Çoban et al. (2017). The adaptation maintained the original number of items but modified the response format from a six-point Likert scale to a five-point Likert scale (1 = Strongly Disagree, 2 = Disagree, 3 = Partially Agree, 4 = Agree, 5 = Strongly Agree). The **total score** on the SCGS ranges from **35 to 175**, with **higher scores** indicating **greater perceived spirituality and spiritual care**.

Subscales and example items:

**Table:** 

Subscale	Example Items
General Characteristics of Spiritual Care	*"Spiritual care requires a trust-based nurse-patient relationship."*
Perspective on Spiritual Care	*"Spirituality helps individuals cope with life’s challenges and problems."*
Definition of Spiritual Care	*"Spiritual care is more comprehensive than religious care."*
Spiritual Care Practices	*"Nurses provide spiritual care by allowing patients to express their fears, anxieties, and concerns."*
Attitudes Toward Spiritual Care	*"I can comfortably provide spiritual care to patients."*

In this study, the SCGS was utilized to assess nursing students' perceptions of spirituality and their attitudes toward spiritual caregiving. The scale consists of 35 items distributed across five subscales, measuring various dimensions of spiritual care. Responses are rated on a five-point Likert scale, with higher scores indicating a more positive perception of spiritual care (İpek Çoban et al., 2017).

### Ethical Considerations

This study was reviewed and approved by the Selçuk University Local Ethics Committee during its meeting on May 24, 2022, and granted ethical approval under decision number 2022/256. Informed consent was obtained from all participants, and permission was obtained from the authors who developed the Peace Scale (PS) and the Spiritual Care-Giving Scale (SCGS). The research protocol and approval procedures adhered to the principles outlined in the Declaration of Helsinki.

## Results

The mean age of the participants was 22.21 ± 1.27 years. The majority of the participants were women, comprising 78.6% of the sample. Most participants came from nuclear families (78.6%), while less than a quarter had extended families (19.3%). A majority of the participants reported a neutral income, where income equaled expenses (77.9%), whereas less than a quarter had a negative income (income less than expenses) (12.1%). More than half of the participants were in their fourth year of study (58.6%), with the remaining being third-year students (41.4%). Over half of the participants had taken art classes in high school (55.7%), and fewer than a quarter had attended extracurricular art courses. Thirty-two participants enrolled in the "Decorative Arts" course for one semester (22.9%), while twenty-two participants took the course for two semesters (15.7%). The details of the participants' descriptive characteristics are provided in Table 1.

**Table 1:** Descriptive characteristics of participants (*n* = 140)

	n	%
*Gender*		
Woman	110	78.6
Men	30	21.4
*Family type*		
Nuclear	110	78.6
Extended	27	19.3
Broken	3	2.1
*Monthly family income*		
Income < expense	17	12.1
Income = expense	109	77.9
Income > expense	14	10.0
*Year (Grade)*		
Third	58	41.4
Fourth	82	58.6
*Did you take an art class in high school?*		
Yes	78	55.7
No	62	44.3
*Do you attend an extracurricular art course?*		
Yes	31	22.1
No	109	77.9
*Have you ever taken the "Decorative Arts" course?*		
Never	86	61.4
For one semester	32	22.9
For two semester	22	15.7

Participants who attended an extracurricular art course (median score = 37.0) had a significantly higher median score on the SCGS 'spirituality perspectives' subscale compared to those who did not attend the course (median score = 35.0) (*p* = 0.045). Additionally, participants who had taken the “Decorative Arts” course for two semesters (median score = 30.0) exhibited significantly higher median scores on the PS scale than those who had never taken the course (median score = 28.0) or had taken it for one semester (median score = 29.0) (*p* = 0.001). Moreover, participants who had taken the “Decorative Arts” course for two semesters demonstrated significantly higher median SCGS subscale scores in 'attributes for spiritual care,' 'spiritual care attitudes,' and 'spiritual care values' than those who had never taken the course (*p* < 0.05) (see Table 2).

**Table 2:** The distribution of SCGS and PS scores by descriptive characteristics

	SCGS						
	PS	Attributes for spiritual care	Spirituality perspectives	Defining spiritual care	Spiritual care attitudes	Spiritual care values	Total SCGS
	Median [IQR]	Median [IQR]	Median [IQR]	Median [IQR]	Median [IQR]	Median [IQR]	Median [IQR]
*Did you take an art class in high school?*							
Yes	28.0(4.0)	42.0(7.2)	36.0(7.0)	31.0(7.0)	31.0(5.0)	17.0(3.0)	156.0(24.7)
No	29.0(3.0)	41.0(8.3)	35.0(7.3)	32.5(7.0)	30.5(5.0)	17.0(3.0)	152.5(28.3)
p* value	.434	.344	.639	.946	.410	.976	.466
*Do you attend an extracurricular art course?*							
Yes	27.0(4.0)	41.0(8.0)	37.0(6.0)	31.0(6.0)	31.0(4.0)	17.0(4.0)	154.0(26.0)
No	28.0(3.5)	41.0(8.0)	35.0(7.0)	32.0(6.5)	31.0(5.0)	17.0(3.0)	156.0(28.0)
p* value	.031	.508	.045	.386	.540	.707	.324
*Have you ever taken the "Decorative Arts" course?*							
Never^(a)^	28.0(4.3)	40.0(7.0)	35.0(7.0)	31.0(7.0)	30.0(4.0)	16.0(2.0)	151.5(25.5)
For one semester^(b)^	29.0(3.8)	41.5(6.8)	36.0(8.0)	30.5(7.0)	31.0(5.7)	17.0(4.0)	152.5(25.3)
For two semesters^(c)^	30.0(4.0)	44.0(3.0)	36.5(7.0)	33.5(4.0)	32.0(3.2)	19.0(2.3)	164.5(19.5)
p** value	.001	< .001	.916	.073	.007	.001	.005
	c > a	c > a			c > a	c > a	c > a

*Kruskal–Wallis Test, **Mann–Whitney U Test**,** *** Kruskal–Wallis test for intergroup comparison

SCGS = Spiritual Care-Giving Scale

PS: Peace Scale

A positive correlation was found between PS and SCGS scores (r = 0.277, *p* = 0.042). Additionally, a strong positive correlation was observed between the SCGS subscale scores for "spiritual care attitudes" and "defining spiritual care" (r = 0.805, *p* < 0.001). Similarly, a positive correlation was noted between the "spiritual care attitudes" and "spiritual care values" subscale scores of the SCGS (r = 0.739, *p* < 0.001) (see Table 3).

**Table 3:** The Correlation between PS and SCGS scores

		PS Total Score	Attributes for Spiritual Care	Spirituality Perspectives	Defining Spiritual Care	Spiritual Care Values	Spiritual Care Attitudes	SCGS Total Score
PS Total Score	r	1	.215	.198	.252	.196	.199	.277^*^
	p		.119	.151	.066	.155	.149	**.042**
Attributes for Spiritual Care	r	.215	1	.122	.355^**^	.343^*^	.406^**^	.734^**^
	p	.119		.381	.008	.011	.002	.000
Spirituality Perspectives	r	.198	.122	1	.723^**^	.665^**^	.685^**^	.696^**^
	p	.151	.381		**< .001**	**< .001**	**< .001**	**< .001**
Defining Spiritual Care	r	.252	.355^**^	.723^**^	1	.821^**^	.805^**^	.844^**^
	p	.066	.008	**< .001**		**< .001**	**< .001**	**< .001**
Spiritual Care Values	r	.196	.343^*^	.665^**^	.821^**^	1	.739^**^	.797^**^
	p	.155	.011	**< .001**	**< .001**		**< .001**	**< .001**
Spiritual Care Attitudes	r	.199	.406^**^	.685^**^	.805^**^	.739^**^	1	.848^**^
	p	.149	.002	**< .001**	**< .001**	**< .001**		**< .001**
SCGS Total Score	r	.277^*^	.734^**^	.696^**^	.844^**^	.797^**^	.848^**^	1
	p	.042	**< .001**	**< .001**	**< .001**	**< .001**	**< .001**	

*Correlation is significant at the 0.05 level (2-tailed). **Correlation is significant at the b. It cannot be computed because at least one of the variables is constant at 0.01 level (2-tailed)

## Discussion

Spirituality is a fundamental aspect of health and well-being, playing a crucial role in holistic care (Momennesab et al., 2019). In recent years, the concept of spirituality has garnered significant attention in various fields, including theology, sociology, psychology, and nursing (Karadağ, 2020). Spirituality extends beyond religion, encompassing philosophical ideas about life (Herlianita et al., 2018). It relates to our qualitative or emotional experiences and offers insight into who we are, why we are, where we come from, and how we wish to be remembered. Spirituality is a collection of wisdom that reflects our life journey and provides answers to the question, “Who am I?” (Makkar & Singh, 2021). Moreover, spirituality enables individuals to better understand themselves, compare their experiences with others, and maintain their self-esteem. It offers hope, strength, and peace of mind, helping individuals cope with challenges (Erişen & Sivrikaya, 2017).

This descriptive study aimed to explore how nursing students perceived peace and spiritual care following their participation in a “Decorative Arts” course.

Regarding sociodemographic characteristics, it is evident that the study had a higher number of female participants. The predominance of female participants is a notable factor to consider when interpreting the results. As the nursing profession is traditionally female-dominated, it is common for the majority of nursing students to be women.

### Peace

The pursuit of peace is a universal desire, with individuals seeking to live free from unease, worry, and anxiety (Öksüz & Karalar, 2019; Çelik, 2020). The definition of peace varies across societies, with Western cultures focusing on individual values and the pursuit of happiness, while Eastern cultures emphasize philosophical and religious traditions. Some argue that happiness is transient, while peace is enduring (Öksüz & Karalar, 2019).

Art has a unique ability to foster a sense of ease, as it resonates with individuals’ emotions and souls. Those who engage with art often find peace or move closer to it. While this may not apply to artists themselves, a significant portion of those involved in art experience tranquility through it. Art embodies vitality, exuberance, serenity, and peace (Ayaydın, 2020).

Our findings show that participants who completed the “Decorative Arts” course over two semesters scored significantly higher on the Peace Scale (PS) compared to those who had never taken the course or participated for only one semester (*p* = 0.001) (Table 2), indicating a clear connection between art and peace. Previous research supports this notion, suggesting that individuals who engage in Sufi rituals (Kayacı & Cengil, 2022) or listen to music (Uygun, 2015) tend to be more peaceful than those who do not. Additionally, people involved in figurative art exhibit greater relaxation and better health outcomes compared to non-participants (Mastandrea et al., 2019). Thus, art is intrinsically linked to peace (Hiçyılmaz & Adanir, 2019). To our knowledge, this study is the first to examine how art influences nursing students’ sense of peace, and as such, it contributes new insights to the existing literature.

The study's sample consisted primarily of female participants, which may influence the results. Women are generally more emotionally expressive than men (Fischer & LaFrance, 2015), and there is a recognized correlation between peace and positive emotions (Demirci & Ekşi, 2017). Therefore, the higher peace levels observed in female students who participated in the arts and crafts course could reflect gender-related preferences or effects.

Spiritual well-being encompasses both peace and life satisfaction (Moberg, 2002). This study posits that both patients and nurses must experience peace of mind to achieve positive health outcomes. Peaceful nursing students are likely to be more receptive to learning, approach their work with greater professionalism, empathize more deeply with patients, and focus on health-promoting interventions. In conclusion, incorporating art courses into nursing curricula may enhance students' sense of peace, thereby benefiting their overall well-being and professional development.

### Spiritual Care Giving

Nurses deliver holistic care to address their patients' needs, with spiritual care being a crucial element of this approach. Spiritual care is considered one of the most effective and essential components of holistic nursing care (Karadağ, 2020; Üzen Cura, 2021). The effectiveness of spiritual care provided by nurses is influenced by various factors, including their perceptions of spirituality and spiritual care, spiritual education, cultural background, and religious beliefs (Herlianita et al., 2018). For nurses to provide effective spiritual care, it is important for them to acknowledge their personal spirituality. Nursing education plays a vital role in this process by enabling students to recognize the spiritual dimension of nursing and increasing their awareness of their own spirituality (Üzen Cura,^2021^).

Participants who had taken the "Decorative Arts" course for two semesters demonstrated significantly higher mean SCGS subscale scores for 'attributes for spiritual care,' 'spiritual care attitudes,' and 'spiritual care values' compared to those who had not taken the course (*p* < 0.05) (Table 2). Although previous research has shown that nurses who engage in group reflection sessions and spiritual care programs develop more positive attitudes toward spirituality and spiritual care, this study is the first to establish a link between art and spirituality. The study sample consisted primarily of women, and the creative expression and artistic activities offered in the "Decorative Arts" course may have provided them with opportunities to express themselves, particularly in the emotional and spiritual realms. This may explain the higher scores of female students on the spiritual care scale.

Lewinson et al. (2015) emphasize that nursing curricula should include spirituality education to help students develop positive attitudes toward spirituality, as many nursing students lack spiritual care competence (Kalkım et al., 2018) and are not skilled in providing spiritual care (Minton et al., 2018; van Leeuwen et al., 2006; Williams et al., 2016). The findings from this study suggest that nursing curricula in Türkiye should consider incorporating art courses to help nursing students adopt a more positive view of spirituality and spiritual caregiving.

Spirituality is inherent in all individuals, often manifesting as inner peace or strength derived from a connection with God or a higher power (Üzen Cura, 2021). It helps individuals understand themselves, compare themselves with others, and maintain their self-esteem. Spirituality provides people with hope, strength, and peace of mind, which are essential for coping with challenges (Erişen & Sivrikaya, 2017).

A positive correlation was found between the Peace Scale (PS) and SCGS scores among participants who took the "Decorative Arts" course (Table 3). In line with this, Çelik (2020) observed that undergraduates commonly use the same language to describe peace, mental health, and well-being. Additionally, Öksüz and Karalar (2019) reported a positive correlation between peace and authenticity, while Demirci and Ekşi (2017) identified a positive relationship between peace and well-being, positive emotions, attachment, meaning, achievement, and health. This study is the first to explore the interconnectedness of art, peace, and spirituality.

While textbooks provide nursing students with a wealth of information on diagnoses, treatments, symptoms, and nursing care, artistic activities offer valuable opportunities for fostering inner peace, developing empathy, and enhancing overall well-being. Engaging with art allows nursing students to better understand the emotional impact of clinical symptoms on patients. Spiritual care primarily aims to reduce patient anxiety, empower them to find inner peace, and alleviate their suffering (Weathers et al., 2016). Nurses who have a positive perception of spiritual care and a high level of peace of mind are better equipped to understand their patients' needs, identify spiritual concerns, and provide care that addresses these needs.

## Conclusions

Nursing students who engage in artistic activities often experience a greater sense of peace and develop a more positive perception of spiritual care. This relationship suggests that integrating art into nursing education can yield significant benefits. To foster these advantages, nursing curricula should incorporate art courses specifically designed for students. Such courses can play a vital role in shaping more favorable attitudes toward spirituality and spiritual caregiving, ultimately contributing to students' overall sense of inner peace and well-being.

These positive transformations can greatly enhance nursing students' ability to empathize with and understand their patients' experiences, both physically and emotionally. Through artistic expression, students may cultivate deeper emotional intelligence, enabling them to connect with their patients on a more profound level. This enhanced understanding allows nursing students to provide more effective and compassionate spiritual care, which is essential in holistic patient treatment.

The findings of this study provide empirical evidence supporting the beneficial effects of art on nursing students' spirituality and mental well-being. The results suggest that nursing students who receive formal education in spirituality and spiritual care are more likely to adopt a holistic approach to patient care. These students develop not only clinical skills but also the compassion and insight needed to address their patients' spiritual needs. Therefore, it is essential for universities to integrate courses focused on spirituality and spiritual caregiving into their nursing programs.

Future research should aim to recruit larger and more diverse sample populations to comprehensively examine the impact of artistic activities on nursing students' peace of mind and spirituality. Such studies will provide a more robust understanding of how these activities influence nursing practice. Additionally, further research should explore the implementation of artistic activities within nursing curricula and evaluate their impact on nurses' spirituality and competence in providing spiritual care. Investigating these aspects will allow educators to develop targeted interventions that enhance both the educational experience and professional readiness of nursing students.

In conclusion, integrating artistic activities into nursing education is not merely an enhancement of the curriculum—it is a crucial step toward nurturing well-rounded healthcare professionals capable of delivering holistic and compassionate care. Such initiatives not only promote nursing students' mental well-being but also improve the quality of care they provide in their future practice.

### Limitation

Participants were included from only one school. Additionally, the majority of participants were women; this may restrict the generalizability of the findings to the broader population of nursing students.

## References

[CR1] Ayaydin, A. (2020). On psychology and art interaction. *Science, Education Art and Technology Journal (SEAT Journal),**4*(1), 8–12. https://dergipark.org.tr/en/pub/bestdergi/issue/50074/569569https://dergipark.org.tr/en/pub/bestdergi/issue/50074/569569

[CR2] Çelik, O. B. (2020). Investigation of tranquility levels of university students in terms of various variables. *International Journal of Contemporary Educational Studies (IntJCES),**6*(1), 233–242.

[CR3] Coban, G. I., Şirin, M., & Yurttaş, A. (2017). Reliability and validity of the spiritual care-giving scale in a Turkish population. *Journal of Religion and Health,**56*(1), 63–73. 10.1007/s10943-016-0261-3 26194169 10.1007/s10943-015-0086-6

[CR4] Demirci, İ, & Ekşi, H. (2017). Development of tranquility scale and examination of its psychometric properties. *Journal of Values Education,**15*(33), 39–60.

[CR5] Erişen, M., & Sivrikaya, S. K. (2017). Spiritual care and nursing. *Gümüşhane University Journal of Health Sciences,**6*(3), 184–190.

[CR6] Fischer, A., & LaFrance, M. (2015). What drives the smile and the tear: Why women are more emotionally expressive than men. *Emotion Review,**7*(1), 22–29. 10.1177/1754073914544406

[CR7] Giske, T., & Cone, P. H. (2015). Discerning the healing path–how nurses assist patient spirituality in diverse health care settings. *Journal of Clinical Nursing,**24*(19–20), 2926–2935. 10.1111/jocn.12907 26215560 10.1111/jocn.12907

[CR8] Gómez-Rincón, C. M. (2023). Art as a spiritual practice: The interplay between artistic creation and spiritual search in seven Colombian artists. *Journal for the Study of Spirituality,**13*(2), 132–146. 10.1080/20440243.2023.2243811

[CR9] Hamid, N., & Dehghanizadeh, Z. (2012). The relationship between spirituality, organizational commitment, and general health with job performance of clinical nurses. *Quarterly Journal of Nursing Management,**1*(2), 20–28.

[CR10] Herlianita, R., Yen, M., Chen, C. H., Fetzer, S. J., & Lin, E. C. L. (2018). Perception of spirituality and spiritual care among Muslim nurses in Indonesia. *Journal of Religion and Health,**57*(2), 762–773. 10.1007/s10943-017-0437-6 28647910 10.1007/s10943-017-0437-6

[CR11] Hiçyılmaz, Y., & Adanır, Y. (2019). Examining, through metaphors, the perceptions of the art term of the prospective teachers who take art education course. *Journal of Social Sciences of Mus Alparslan University,**7*(3), 19–23. 10.18506/anemon.462012

[CR116] Huet, V. (2016). Case study of an art therapy-based group for work-related stress with hospice staff. *International Journal of Art Therapy,**22*(1), 22–34. 10.1080/17454832.2016.1260039

[CR118] İpek Çoban, G., Şirin, M., & Yurttaş, A. (2017). Reliability and validity of the Spiritual Care-Giving Scale in a Turkish population. *Journal of Religion and Health,**56*(1), 63–73. 10.1007/s10943-015-0086-6 26194169 10.1007/s10943-015-0086-6

[CR12] Kaddourah, B., Abu-Shaheen, A., & Al-Tannir, M. (2018). Nurses’ perceptions of spirituality and spiritual care at five tertiary care hospitals in Riyadh, Saudi Arabia: A cross-sectional study. *Oman Medical Journal,**33*(2), 154. 10.5001/omj.2018.28 29657685 10.5001/omj.2018.28 PMC5889832

[CR13] Kalkim, A., SagkalMidilli, T., & Daghan, S. (2018). Nursing students’ perceptions of spirituality and spiritual care and their spiritual care competencies: A correlational research study. *Journal of Hospice and Palliative Nursing,**20*(3), 286–295. 10.1097/NJH.0000000000000446 30063680 10.1097/NJH.0000000000000446

[CR14] Karadağ, E. (2020). Do perceptions of spiritual care affect attitudes towards care for dying patients in a group of Turkish nursing students? *Journal of Religion and Health,**59*(4), 1702–1712. 10.1007/s10943-019-00815-9 30972609 10.1007/s10943-019-00815-9

[CR15] Kayacı, E., & Cengil, M. (2022). An empirical research on the relationship between moral maturity and peace in Sufis. *Journal of Kocatepe Islamic Sciences,**5*(1), 12–31.

[CR16] Koenig, H. G., & Al Zaben, F. (2021). Psychometric validation and translation of religious and spiritual measures. *Journal of Religion and Health,**60*(5), 3467–3483. 10.1007/s10943-021-01373-9 34331172 10.1007/s10943-021-01373-9

[CR17] Kroning, M. (2018). Student perceptions of spirituality and spiritual care. *Journal of Christian Nursing,**35*(2), E17–E20. 10.1097/CNJ.0000000000000490 29521915 10.1097/CNJ.0000000000000490

[CR18] Lewinson, L. P., McSherry, W., & Kevern, P. (2015). Spirituality in pre-registration nurse education and practice: A review of the literature. *Nurse Education Today,**35*(6), 806–814. 10.1016/j.nedt.2015.01.011 25707759 10.1016/j.nedt.2015.01.011

[CR19] Makkar, S., & Singh, A. K. (2021). Development of a spirituality measurement scale. *Current Psychology,**40*, 1490–1497. 10.1007/s12144-018-0081-7

[CR20] Mastandrea, S., Fagioli, S., & Biasi, V. (2019). Art and psychological well-being: Linking the brain to the aesthetic emotion. *Frontiers in Psychology,**10*, 739. 10.3389/fpsyg.2019.00739 31019480 10.3389/fpsyg.2019.00739 PMC6458291

[CR21] Minton, M. E., Isaacson, M. J., Varilek, B. M., Stadick, J. L., & O’Connell-Persaud, S. (2018). A willingness to go there: Nurses and spiritual care. *Journal of Clinical Nursing,**27*(1–2), 173–181. 10.1111/jocn.13867 28474751 10.1111/jocn.13867

[CR22] Moberg, D. O. (2002). Assessing and measuring spirituality: Confronting dilemmas of universal and particular evaluative criteria. *Journal of Adult Development,**9*(1), 47–60. 10.1023/A:1013877201375

[CR119] Momennasab, M., Shadfard, Z., Jaberi, A., Najafi, S. S., & Hosseini, F. N. (2019). The effect of group reflection on nursing students' spiritual well-being and attitude toward spiritual care: A randomized controlled trial. *Investigación y Educación en Enfermería,**37*(1), e09. 10.17533/udea.iee.v37n1e09 31083846 10.17533/udea.iee.v37n1e09 PMC7871466

[CR104] Musa, A. S. (2017). Spiritual care intervention and spiritual well-being. *Journal of Holistic Nursing,**35*(1), 53–61. 10.1177/0898010116644388 27105890 10.1177/0898010116644388

[CR120] Öksüz, Y., & Karalar, M. (2019). The relationship between tranquility and authenticity levels of university students. *The Journal of Education, Theory and Practical Research,**5*(3), 321–336. https://dergipark.org.tr/tr/pub/ekuad/issue/51148/666648

[CR103] Pesut, B. (2008). Spirituality and spiritual care in nursing fundamentals textbooks. *The Journal of Nursing Education,**47*(4),167–173. 10.3928/01484834-20080401-05 18468293 10.3928/01484834-20080401-05

[CR115] Potash, J. S., Chen, J. Y., Lam, C. L., & Chau, V. T. (2014). Art-making in a family medicine clerkship: How does it affect medical student empathy?. *BMC Medical Education,**14*, 1–9. 10.1186/s12909-014-0177-9 25431323 10.1186/s12909-014-0247-4 PMC4256925

[CR102] Ross, L., van Leeuwen, R., Baldacchino, D., Giske, T., McSherry, W., Narayanasamy, A., Downes, C., Jarvis, P., & Schep- Akkerman, A. (2014). Student nurses’ perceptions of spirituality and competence in delivering spiritual care: A European pilot study. *Nurse Education Today,**34*(5), 697–702. 10.1016/j.nedt.2013.09.014 24119953 10.1016/j.nedt.2013.09.014

[CR113] Ross, L., Giske, T., van Leeuwen, R., Baldacchino, D., McSherry, W., Narayanasamy, A., Jarvis, P., & Schep-Akkerman, A. (2016). Factors contributing to student nurses' / midwives' perceived competency in spiritual care. *Nurse Education Today,**36*, 445–451. 10.1016/j.nedt.2015.10.005 26541988 10.1016/j.nedt.2015.10.005

[CR100] Ross, L., McSherry, W., Giske, T., van Leeuwen, R., Schep-Akkerman, A., Koslander, T., Hall, J., Steenfeldt, V. Ø., & Jarvis, P. (2018). Nursing and midwifery students' perceptions of spirituality, spiritual care, and spiritual care competency: A prospective, longitudinal, correlational European study. *Nurse Education Today,**67*, 64–71. 10.1016/j.nedt.2018.05.002 29763841 10.1016/j.nedt.2018.05.002

[CR114] Sarman, A., & Günay, U. (2023). The effect of calligraphy as an art therapy intervention containing religious motifs, on the anxiety and depression in adolescent psychiatric patients. *Journal of Religion and Health,**62*(2), 1269–1285. 10.1007/s10943-021-01479-0 35059964 10.1007/s10943-021-01479-0

[CR101] Selman, L. E., Brighton, L. J., Sinclair, S., Karvinen, I., Egan, R., Speck, P., Powell, R. A., Deskur-Smielecka, E., Glajchen, M., Adler, S., Puchalski, C., Hunter, J., Gikaara, N., Hope, J., & InSpirit Collaborative. (2018). Patients' and caregivers' needs, experiences, preferences and research priorities in spiritual care: A focus group study across nine countries. *Palliative Medicine,**32*(1), 216–230. 10.1177/0269216317734954 29020846 10.1177/0269216317734954 PMC5758929

[CR112] Smith, A. P. B., & Read, J. E. (2017). Art, objects, and beautiful stories: A “new” approach to spiritual care. *Journal of Pastoral Care & Counseling,**71*(2), 91–97. 10.1177/1542305017703126 28618884 10.1177/1542305017703126

[CR117] Tiew, L. H., & Creedy, D. K. (2012). Development and preliminary validation of a composite Spiritual Care-Giving Scale. *International Journal of Nursing Studies,**49*(6), 682–690. 10.1016/j.ijnurstu.2011.11.014 22197053 10.1016/j.ijnurstu.2011.11.014

[CR110] Timmins, F., & Caldeira, S. (2017). Understanding spirituality and spiritual care in nursing. *Nursing Standard,**31*(22),50–57. 10.7748/ns.2017.e10311 28120672 10.7748/ns.2017.e10311

[CR121] Üzen Cura, Ş. (2021). Nursing students' spiritual orientations and their attitudes toward the principles of dying with dignity: A sample from Turkey. *Journal of Religion and Health,**60*(1), 221–231. 10.1007/s10943-020-01029-0 32418151 10.1007/s10943-020-01029-0

[CR111] van Leeuwen, R., Tiesinga, L. J., Post, D., & Jochemsen, H. (2006). Spiritual care: Implications for nurses' professional responsibility. *Journal of Clinical Nursing,**15*(7), 875–884. 10.1111/j.1365-2702.2006.01615.x 16879380 10.1111/j.1365-2702.2006.01615.x

[CR23] Weathers, E., McCarthy, G., & Coffey, A. (2016). Concept analysis of spirituality: An evolutionary approach. *Nursing Forum,**51*(2), 79–96. 10.1111/nuf.12128 25644366 10.1111/nuf.12128

[CR24] Williams, M. G., Voss, A., Vahle, B., & Capp, S. (2016). Clinical nursing education: Using the FICA spiritual history tool to assess patients’ spirituality. *Nurse Educator,**41*(4), E6–E9. 10.1097/NNE.0000000000000269 27166591 10.1097/NNE.0000000000000269

[CR25] Zare, A., & Jahandideh, S. (2014). The impact of special wards nursing spiritual well-being upon patients’ spiritual care. *Iranian Journal of Nursing Research,**9*(3), 30–38.

